# How Adhesion Molecule Patterns Change While Neutrophils Traffic through the Lung during Inflammation

**DOI:** 10.1155/2019/1208086

**Published:** 2019-02-28

**Authors:** Franziska M. Konrad, Julia Wohlert, Jutta Gamper-Tsigaras, Kristian-Christos Ngamsri, Jörg Reutershan

**Affiliations:** ^1^Department of Anesthesiology and Intensive Care Medicine, University Hospital of Tübingen, Tübingen, Germany; ^2^Department of Anesthesiology and Intensive Care Medicine, Hospital of Bayreuth, Germany

## Abstract

In acute pulmonary inflammation, polymorphonuclear cells (PMNs) pass a transendothelial barrier from the circulation into the lung interstitium followed by a transepithelial migration into the alveolar space. These migration steps are regulated differentially by a concept of adhesion molecules and remain—despite decades of research—incompletely understood. Current knowledge of changes in the expression pattern of adhesion molecules mainly derives from in vitro studies or from studies in extrapulmonary organ systems, where regulation of adhesion molecules differs significantly. In a murine model of lung inflammation, we determined the expression pattern of nine relevant neutrophilic adhesion molecules on their way through the different compartments of the lung. We used a flow cytometry-based technique that allowed describing spatial distribution of the adhesion molecules expressed on PMNs during their migration through the lung in detail. For example, the highest expression of CD29 was found in the intravascular compartment, highlighting its impact on the initial adhesion to the endothelium. CD47 showed its peak of expression on the later phase of transendothelial migration, whereas CD11b and CD54 expression peaked interstitial. A pivotal role for transepithelial migration was found for the adhesion molecule CD172a. Thereby, expression may correlate with functional impact for specific migration steps. In vitro studies further confirmed our in vivo findings. In conclusion, we are the first to determine the changes in expression patterns of relevant adhesion molecules on their migration through the different compartments of the lung. These findings may help to further understand the regulation of neutrophil trafficking in the lung.

## 1. Introduction

In pulmonary inflammation, chemotactic factors are released by macrophages and pulmonary epithelial and endothelial cells [1–4] to recruit polymorphonuclear neutrophils (PMNs)—as the first leukocytes—to sites of inflammation. On their way of migration, PMNs have to pass an endothelial barrier from the circulation into the lung interstitium, followed by an epithelial barrier into the alveolar space. These migration steps are regulated differently and tightly by the interaction of multiple molecules, expressed on both hematopoietic and nonhematopoietic cells, typically referred to as adhesion cascade.

Since accumulation of PMNs in the lung interstitium and alveolar space are considered a key characteristic of acute pulmonary inflammation [5], current research demands further studies on PMN trafficking into tissue during inflammation [6]. Especially, the neutrophil recruitment cascade in the lung remains mainly elusive [7]. We therefore investigated the expression of distinct adhesion molecules on PMNs on their migratory way through the different compartments of the lung—intravascular, adherent to the pulmonary endothelium, in the lung interstitium and alveolar space to provide further inside into the physiological mechanisms of PMN trafficking through the inflamed lungs.

Therefore, we determined the frequency of parents (meaning the percentage of PMNs expressing the adhesion molecule in relation to all PMNs) and the mean fluorescence intensity (MFI; how much of the adhesion molecule is expressed on each single PMN) of the investigated nine neutrophilic adhesion molecules, which are known to play a detrimental role in PMN migration. We focused on the expression pattern rather than on the interaction of the different adhesion molecules; this should be investigated in further investigations.

The findings of the presented study complement the existing data and, for the first time, put light on the complete migration cascade of neutrophils throughout the lung including transendothelial and transepithelial migration.

## 2. Materials And Methods

### 2.1. Animals

We used C57BL/6 male wild-type mice from Charles River Laboratories (Germany), between 8 and 12 weeks old. All animal protocols were approved by the Animal Care and Use Committee of the University of Tübingen.

### 2.2. Murine Model of Acute Lung Injury

As described in detail before, 4 to 8 animals inhaled nebulized LPS (Salmonella enteritidis; Sigma–Aldrich; 7 ml in total; 500 *μ*g/ml), which led to a reproducible acute pulmonary inflammation with migration of PMNs into the different compartments of the lung (intravascular, adherent to the endothelium, interstitial, intra-alveolar), an increase of chemokines and microvascular permeability [8, 9]. Control mice inhaled the solvent NaCl 0.9%.

### 2.3. Gene Expression of Adhesion Molecules

Total RNA was isolated from murine lungs 3 h after LPS inhalation by using peqGOLD TriFast™ (Peqlab, Germany) and cDNA synthesis was performed by Bio-Rad iScript kit (Bio-Rad, Germany) according to the manufacturer's direction.

We determined the expression of different adhesion molecules in the lungs of mice by RT-PCR [8]. This method was performed with the following primers for CD11a (5′-agatcgagtccggacccacag-3′ and 5′-ggcagtgaagaggcctcccg-3′), CD11b (5′-atggacgctgatggcaatacc-3′ and 5′-tccccattcacgtctccca-3′), CD31 (5′-ccaaggccaaacaga -3′ and 5′-aagggagccttccgttct-3′), CD54 (5′-caatttctcatgccgcacag-3′ and 5′-agctggaagatcgaaagtccg-3′), CD162 (5′-tttgctagctccactctcac-3′ and 5′-atgtttattggatgcctgtgc-3′), CD47 (5′-catcgtggttgttggagccatc-3′ and 5′-acagtccgtcacttcccttcac-3′), CD172a (5′-agtccaccttaagaggaccaagtagc-3′ and 5′-tgtacagaaacaggacgcgga-3′), CD44 (5′-cgcacaccatggacaagttt-3′ and 5′-gaaagccttgcagaggtcag-3′), CD29 (5′-gccagggctggttatacaga-3′ and 5′-tcacaatggcacacaggttt-3′), and 18S as housekeeping gene (5′-gtaacccgttgaaccccatt-3′ and 5′-ccatccaatcggtagtagcg-3′).

### 2.4. Immunofluorescence Images

The lungs were embedded in paraffin (*n* = 2) as described before [10]. Lung sections were fixed for 10 minutes in acetone and methanol. After washing, lung sections were permeabilized with 1% TritonX and blocked with 5% BSA in PBS for 1 h. Sections were stained with rabbit anti-CD29 (Sc-7884, Santa Cruz, Dallas, USA), goat anti-CD54 (Sc-1511, Santa Cruz), goat anti-CD31 (Sc-1506, Santa Cruz, Dallas, USA), rat anti-CD31 (Sc-101454, Santa Cruz), and rat anti-Ly6b (MCA771GA, AbD Serotec, Kidlington, UK). For visualization, the following secondary antibodies were employed: donkey anti-rabbit IgG Alexa Fluor 488 (Sc-362261, Santa Cruz), goat anti-rabbit IgG Alexa Fluor 488 (A11008, Thermo Fisher Scientific), donkey anti-goat IgG Alexa Fluor 594 (A-11058, Life Technologies, Oregon, USA), and chicken anti-rat IgG Alexa Fluor 594 (A-21471, Life Technologies). For nuclei counterstaining, we used Roti-Mount FluorCare DAPI (HP20.1, Carl Roth, Germany). For IgG-controls, see Supplemental Figure 1.

### 2.5. In Vivo Migration Assay

24 h after LPS inhalation, we determined PMN migration into the different compartments of the lung via a flow cytometry-based method as described in detail before [11]. The time point was chosen based on our previously performed time courses with the peak of PMN migration into the alveolar space after 24 h [11, 12]. Briefly, fluorescent GR-1 (clone RB6-8C5) was injected into the tail vein of mice to mark all intravascular PMNs. We performed a thoracotomy in deep anesthesia, injected 2 ml of saline into the beating heart to flush the lungs free of blood and nonadherent leukocytes. Tracheal incision was conducted and 2 ml PBS was installed to harvest the bronchoalveolar lavage (BAL). The lungs were incubated with an enzymatic cocktail (DNAse, collagenase, and hyaluronidase) for 30 minutes at 37°C, homogenized by using a cell strainer, lysed centrifuged, and resuspended in staining buffer. In the following, cells were incubated with fluorescent antibodies against CD45 (clone 30-F11) and 7/4 (clone 7/4). Intravascular PMNs were now identified as CD45+, 7/4+, and GR-1+; whereas interstitial PMNs were assigned as CD45+, 7/4+, and GR-1 negative cells. Absolute cell counts were determined in the BAL and lungs. The detailed description of the method has been described elsewhere [11].

For the detection of the adhesion molecules, the following specific fluorescent antibodies were added to cell suspensions: CD11a (catalog number 553121, BD, Heidelberg, Germany), CD11b (553311, BD), CD31 (553373, BD), CD54 (A16333, Life Technologies, Frederick, USA), CD162 (555306, BD), CD47 (563585, BD), CD172a (144011, BioLegend, San Diego, CA, USA), CD44 (553134, BD), and CD29 (25-0291-80 eBioscience, San Diego, USA). As corresponding IgG controls were IgG1 (345816, BD), IgG2a (553929, BD; 349053, BD), and IgG2b (553991, BD; 0302B, AbD Serotec, Kidlington, UK) used. For the gating process see supplemental Figure 2.

### 2.6. In Vitro PMN Migration

Human epithelial cells were cultivated on inserts of a Transwell system (Costar, MA, USA) until reaching confluence. Thereby, cells were seeded on the bottom side of the inserts, so that cells migrate—comparable to the in vivo migration—from the basolateral side through the epithelial monolayer. PMNs from whole human blood migrated through a monolayer of epithelial cells along a chemotactic gradient (fMLP; 20 ng/ml; Sigma-Aldrich, Steinheim, Germany) (Supplemental Figure 3). After 4 h, PMNs in the upper chamber and also migrated PMNs in the lower chamber were collected. To determine PMNs in the epithelial layer, wells were treated with 800 *μ*l Accutase and incubated and washed with 2 ml medium. Absolute cell counts were determined. PMNs were identified CD45 (B183057, BioLegend) and CD66b (E16847-109 eBioscience) positive, adhesion molecules by: CD11a (catalog number 3189709, BD), CD11b (54342, BD), CD31 (4065855, BD), CD54 (4301917, BD), CD162 (61514, BD), CD47 (E15361-103, eBioscience), CD172a (3290897, BD), CD44 (B180554, BioLegend), and CD29 (4262918, BD). As corresponding IgG controls were IgG1 (16651, BD) and IgM (E13114-104, eBioscience) used.

### 2.7. Statistical Analysis

Data are presented as mean ± SD unless indicated otherwise. Statistical analysis was performed using GraphPad Prism version 5.3 for Windows (GraphPad Software, San Diego, CA, USA). Differences between the groups were evaluated by one-way ANOVA followed by Bonferroni post hoc test or Student *t*-test to compare 2 groups. *P* < 0.05 was considered statistically significant.

## 3. Results

### 3.1. LPS-Induced PMN Migration

**Figure 1:**
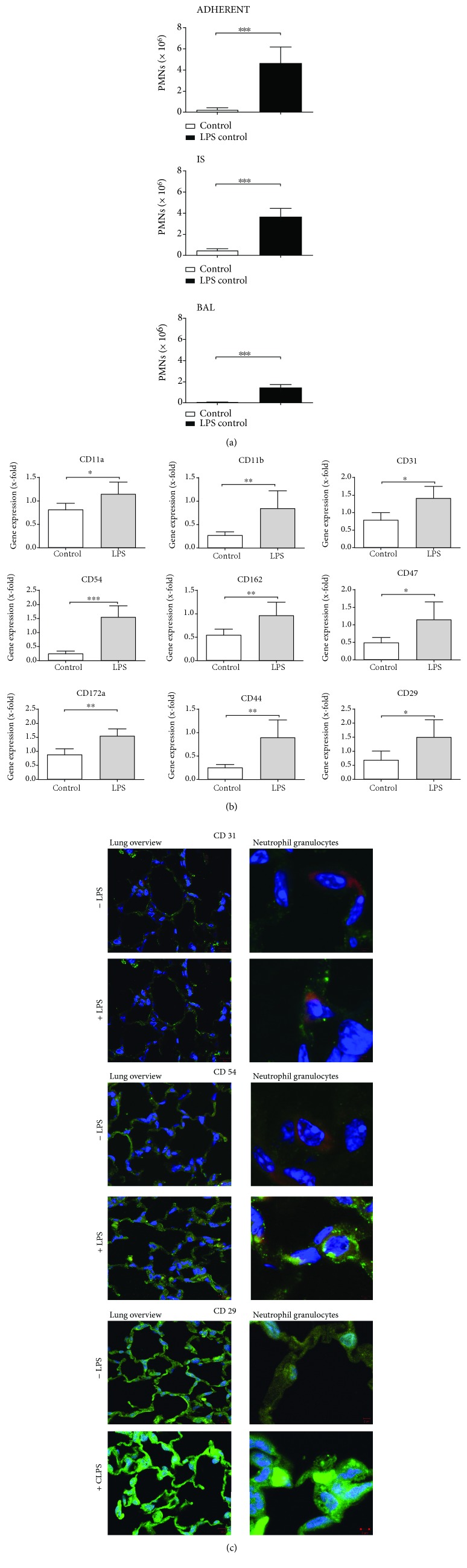
Migration of polymorphonuclear neutrophils (PMNs) into the different compartments of the lung (a). LPS inhalation caused an increase of PMNs adherent to the pulmonary endothelium (ADHERENT), in the lung interstitium (IS), and the alveolar space (BAL) after the inflammation (*n* = 8 − 12). Impact of inflammation on the expression of adhesion molecules in the lungs of mice (b). The expression of the adhesion molecules were investigated after the onset of inflammation by RT-PCR (*n* = 4 − 8). Data are presented as mean ± SD; ^∗^ *P* < 0.05; ^∗∗^ *P* < 0.01; ^∗∗∗^ *P* < 0.001 as indicated. Determination of adhesion molecule expression by immunofluorescence (c). Mice were treated with LPS and three representative adhesion molecules investigated (*n* = 4). Images are representatives of four experiments with similar results (original magnification, ×63). The particular adhesion molecule was stained with a specific antibody and appears green, nuclei were stained with DAPI and emerge blue, and polymorphonuclear leukocytes (PMN) appear red.

24 h after LPS inhalation, PMNs adherent to the pulmonary endothelium were significantly increased (Figure 1(a)). Also, PMN influx into the lung interstitium was evident as was PMN accumulation in the alveolar space. This time point was chosen based on our experience on PMN migration in acute pulmonary inflammation, where PMNs need a certain amount of time to migrate significantly into the alveolar space [8, 11].

### 3.2. Expression of Adhesion Molecules in the Lungs of Mice

Gene expression of all nine investigated adhesion molecules—CD11a, CD11b, CD44, CD162, CD29, CD54, CD47, CD31, and CD172a—was significantly increased in whole lung tissue of mice after the onset of inflammation (Figure 1(b)).

Protein expression of selected adhesion molecules in the lungs of mice was investigated by immunofluorescence (Figure 1(c)). After LPS inhalation, the expression of CD31 increased in the lung and also on PMNs that had been migrated into the lung. These enhancements of the protein expression of the adhesion molecule after the inflammation were also found by staining CD54 and CD29. The expression of both molecules was increased after LPS in the lung and also on migrated PMNs.

Since this technique did not allow a further discrimination of the expression pattern of the distinct adhesion molecules in the different compartments of the lung, we used a flow cytometry-based method to investigate them in detail.

### 3.3. Adhesion Molecules on PMNs

24 h after the induction of inflammation, the expression pattern of migrated PMNs into the lung was further differentiated. At this time point, our model reflects acute pulmonary inflammation with migration of PMNs into the BAL [11].

#### 3.3.1. CD11a (Lymphocyte Function-Associated Antigen 1; LFA-1)

**Figure 2:**
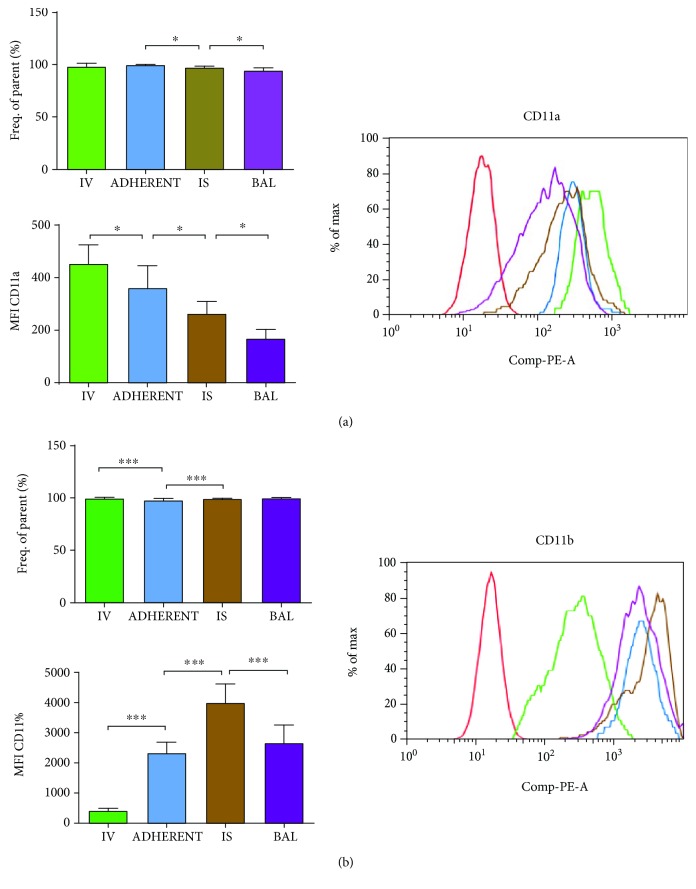
Expression of CD11a (a) and CD11b (b) on PMNs in the different compartments of the lung. Mice were treated with LPS, and after 24 h, PMNs expressing CD11b on their surface were identified (% of frequency of parents) in the different compartments of the lung (IV = intravascular; ADHERENT = adherent to the endothelium; IS = interstitial; BAL = bronchoalveolar lavage). Next, the molecule expression of the adhesion molecule per PMN was analyzed by the determination of the mean fluorescence intensity (MFI). One representative histogram of the different MFIs of the adhesion molecule on PMNs in the different compartments of the lung is shown, whereas the red histogram resembles the negative control. Data are presented as mean ± SD; *n* = 8 − 10 (a); *n* = 10 − 16 (b); ^∗^ *P* < 0.05; ^∗∗^ *P* < 0.01; ^∗∗∗^ *P* < 0.001 as indicated.

PMNs in the intravascular system—either circulating or adherent to the endothelium—had the highest percentage of PMNs expressing CD11a on their surface (displayed as “frequency of parent” and therefore not depending on cell counts, Figure 2(a)). After the migration into the lung interstitium, a significant reduction took place, and the expression of CD11a was again significantly diminished after the migration into the BAL.

Next, we investigated the surface expression of CD11a on each PMN (MFI = mean fluorescence intensity, see Supplemental Figure 4 for further explanation). The surface expression of CD11a on PMNs on their way through the different compartment of the lung was already significantly reduced after the circulating PMNs attached to the endothelium. PMNs migrated into the lung interstitium showed a further significant decrease, which continued after PMNs where migrated into the alveolar system.

Taken together, both—the percentage of PMNs expressing CD11a and the amount of CD11a expressed on each PMN—decreased significantly through the migration of PMNs through the different compartments of the lung. This highlights the impact of the adhesion molecule CD11a on the intravascular compartment.

#### 3.3.2. CD11b (Macrophage-1; MAC-1)

The fraction of CD11b-positive PMNs of the particular compartment of the lung was significantly reduced after PMNs attached to the endothelium (Figure 2(b)). After the migration into the lung interstitium, the contingent of CD11b-positive PMNs rose significantly and stayed at this level in the alveolar space.

The expression of CD11b on the PMN surface rose significantly, when PMNs were attached to the endothelium and rose further after the migration into the interstitium. PMNs in the BAL showed a significantly lower expression compared to PMN in the lung interstitium, indicating that the expression of CD11b is important for the adhesion to the endothelium and especially for the further interstitial migration.

#### 3.3.3. CD31

**Figure 3:**
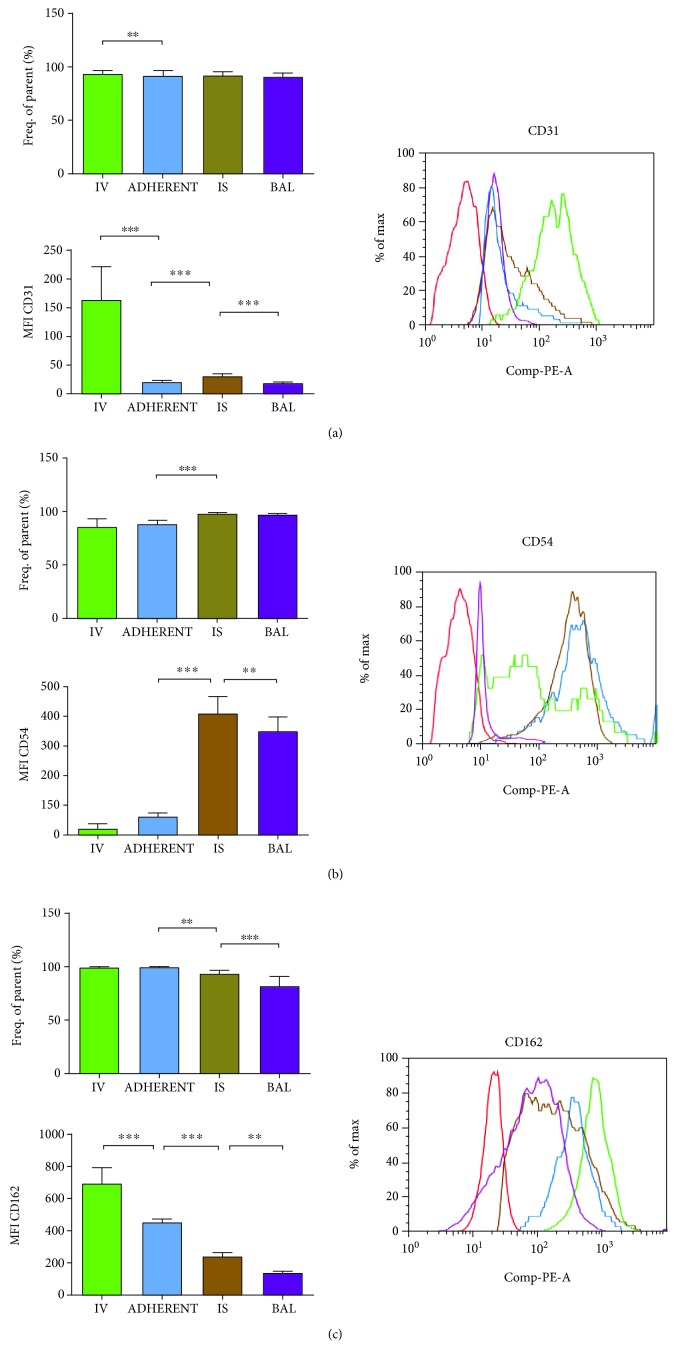
Impact of inflammation on the expression of CD31 (a), CD54 (b), and CD162 (c) on PMNs in the different compartments of the lung. All mice inhaled LPS and the fraction of PMNs which express the particular adhesion molecule on their surface was determined (% of frequency of parents) in the different compartments of the lung (IV = intravascular; ADHERENT = adherent to the endothelium; IS = interstitial; BAL = bronchoalveolar lavage). Additionally, the mean fluorescence intensity (MFI) was analyzed for the PMNs in each compartment and one representative histogram is shown, whereas the red histogram resembles the negative control. Data are presented as mean ± SD; *n* = 8 − 12 (a–c); ^∗^ *P* < 0.05; ^∗∗^ *P* < 0.01; ^∗∗∗^ *P* < 0.001 as indicated.

A high percentage of the PMNs expressed CD31 throughout the different compartments of the lung, with no differences in between (Figure 3(a)).

The platelet endothelial cell adhesion molecule (PECAM, CD31) had its highest expression on intravascular PMNs and was significantly reduced after PMNs attached to the endothelium. Migration into the lung interstitium increased the expression again significantly, whereas it was again significantly diminished after transepithelial migration into the BAL.

#### 3.3.4. CD54

CD54 was expressed by over 85% of PMNs in the intravascular compartment and increased significantly after the transendothelial migration into the interstitium of the lung and remained elevated after transepithelial migration (Figure 3(b)).

These findings were further reflected by the denseness of the expression of the adhesion molecule on PMNs. CD54 was expressed on a rather low level on circulating PMNs but increased detrimental after migration into the lung interstitium, where CD54 had its peak; but it was slightly reduced on PMNs in the BAL again.

#### 3.3.5. CD162

P-Selectin glycoprotein ligand-1 (PSGL-1, CD162) was expressed by nearly all PMNs intravascular—circulating and adherent to the endothelium (Figure 3(c)). After passing the endothelial barrier, significantly less PMNs showed CD162 on their surface and this expression was further reduced after passing the alveolar epithelium.

The determination of the amount of CD162 on PMNs further classified the importance of the adhesion molecule in the circulation. After PMNs adhered to the endothelium, their expression of CD162 was already significantly reduced. This decrease was continued after each migration step—into the lung interstitium and the alveolar system, highlighting the importance of CD162 on PMNs for the initial PMN/endothelium contact.

#### 3.3.6. CD47

**Figure 4:**
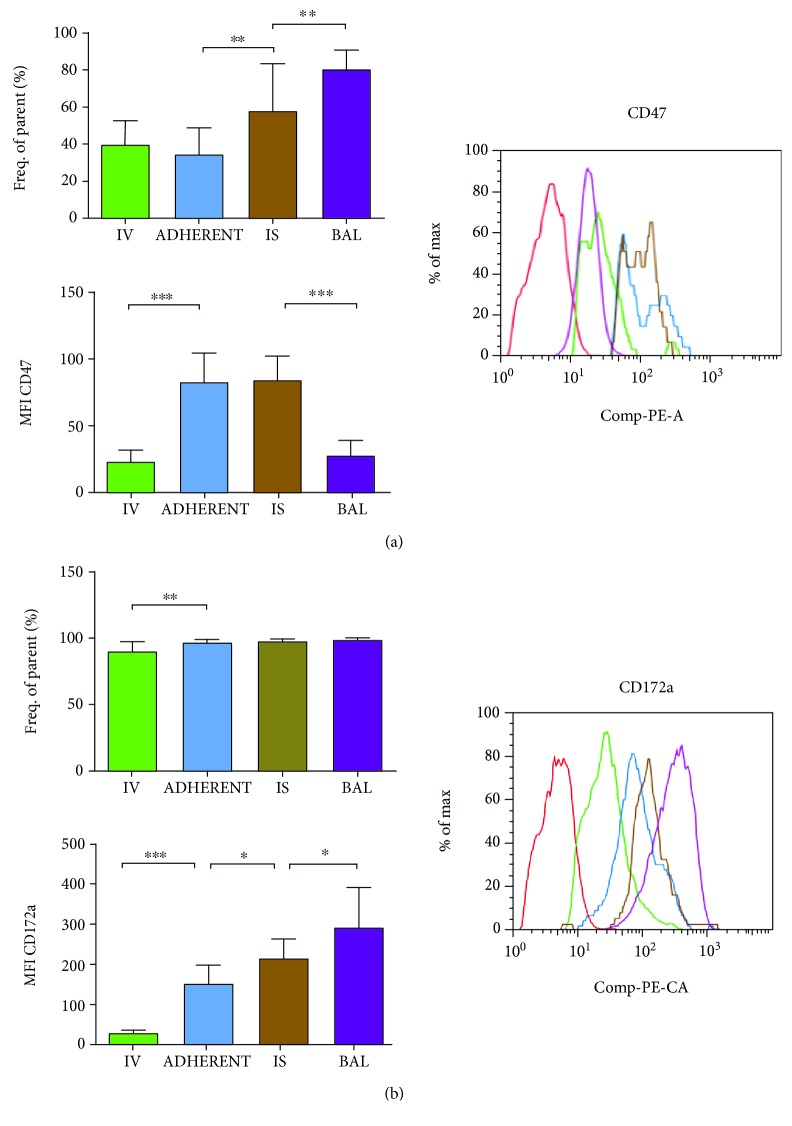
Expression of CD47 (a) and CD172a (b) on PMNs during migration through the lung. An inflammation was induced in all mice, and 24 h later, the frequency of parents of the adhesion molecules was investigated in the different compartments of the lung (IV = intravascular; ADHERENT = adherent to the endothelium; IS = interstitial; BAL = bronchoalveolar lavage). To assess the amount of molecules on each PMN, the mean fluorescence intensity (MFI) was analyzed and one representative histogram is shown, whereas the red histogram resembles the negative control. Data are presented as mean ± SD; *n* = 8 − 16 (a, b); ^∗^ *P* < 0.05; ^∗∗^ *P* < 0.01; ^∗∗∗^ *P* < 0.001 as indicated.

In contrast, CD47 is only expressed by 35–40% of the PMNs intravascular (Figure 4(a)). After migration into the lung interstitium, the amount of CD47-positive PMNs increased significantly and a further augmentation was detectable in the alveolar space.

The surface expression of the integrin-associated protein (IAP, CD47) increased significantly after the adhesion to the endothelium, stayed elevated in the lung interstitium, and was significantly reduced after the transepithelial migration into the alveolar space—pointing out its importance for the transendothelial and interstitial migration.

#### 3.3.7. CD172a

The percentage of CD172a-positive PMNs increased significantly after PMNs attached to the endothelium and stayed elevated in the following compartments of the lung (Figure 4(b)).

Concordantly, the level of signal-regulatory protein alpha (SIRP*α*, CD172a) accelerated significantly after PMNs had contact to the endothelium but also after the migration step into the lung interstitium and had its peak of expression after transepithelial migration into the alveolar space.

#### 3.3.8. CD44 (HCAM, Homing Cell Adhesion Molecule)

**Figure 5:**
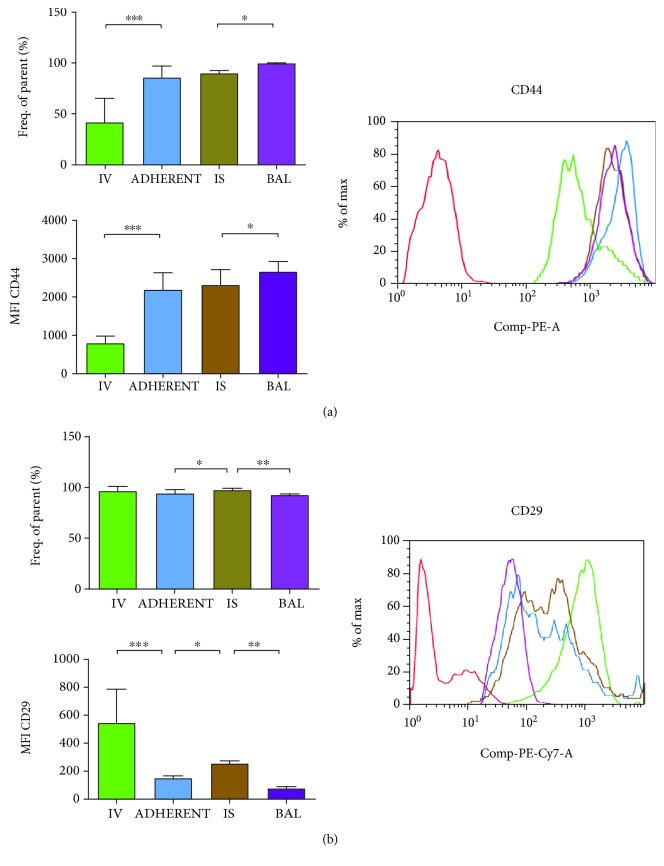
Distribution of the adhesion molecules CD44 (a) and CD29 (b) on PMNs migrating through the different compartments of the lung. In the different compartments of the lung (IV = intravascular; ADHERENT = adherent to the endothelium; IS = interstitial; BAL = bronchoalveolar lavage) were the numbers of PMNs assessed that express the particular adhesion molecules (frequency of parents). By using the mean fluorescence intensity (MFI), the total amount of molecules on each PMN was evaluated and one representative histogram is shown, whereas the red histogram resembles the negative control. Data are presented as mean ± SD; *n* = 7 − 13 (a, b); ^∗^ *P* < 0.05; ^∗∗^ *P* < 0.01; ^∗∗∗^ *P* < 0.001 as indicated.

The frequency of CD44-positive PMNs significantly augmented after contact with the endothelium, stayed elevated interstitial, and increased even further after reaching the bronchoalveolar system (Figure 5(a)).

CD44 is an adhesion molecule, which was expressed at high levels on PMNs after they adhered to the pulmonary endothelium. CD44 expression on PMNs stayed elevated interstitial and increased even further after transepithelial migration into the alveolar space.

#### 3.3.9. CD29

Over 90% of the circulating PMNs showed CD29 on their surface, the percentage increased even significantly after migrating into the lung interstitium and decreased again on PMNs in the alveolar space (Figure 5(b)).

Therefore, the amount of CD29 on PMNs had its highest expression intravascular and was already significantly reduced after PMNs attached to the endothelium. The transendothelial migration into the interstitium increased the surface expression of CD29 again, whereas the lowest expression was found on PMNs in the BAL.

### 3.4. In Vitro Transmigration

**Figure 6:**
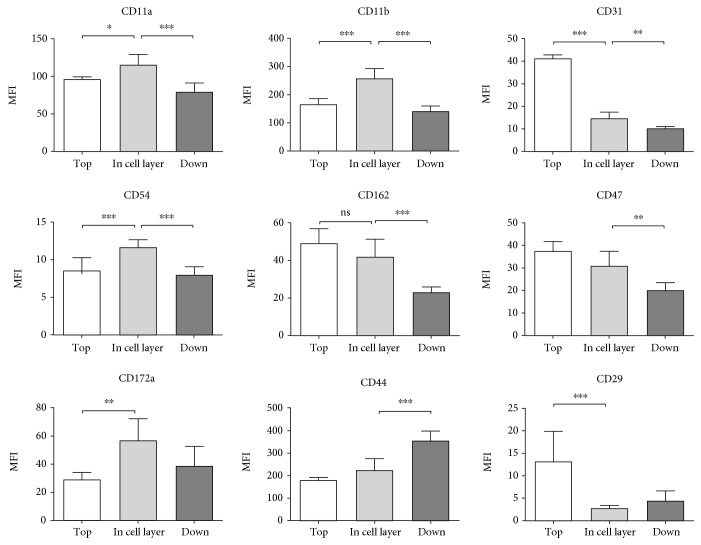
Investigating the adhesion molecules in vitro. In vitro transmigration assay of human PMNs through a pulmonary epithelial monolayer. Migration of PMNs was initiated through fMLP and PMNs in the upper chamber, in the epithelial layer and in the bottom well relating to their expression of adhesion molecules investigated (*n* = 4 − 10). Data are presented as mean ± SD; ^∗^ *P* < 0.05; ^∗∗^ *P* < 0.01; ^∗∗∗^ *P* < 0.001 as indicated.

To verify our results from the in vivo migration assay, we performed in vitro transmigration assays, where PMNs from human blood migrated through a monolayer of pulmonary epithelial cells induced by fMLP (Figure 6). We studied transepithelial migration since this is the underinvestigated migration step in the lung. PMNs were identified by flow cytometry. The main fraction of PMNs did not migrate, a small amount was found in the cell layer and the third fraction resembled the migrated PMNs.

Completely in line with our findings from the in vivo migration assay and therefore confirming our results were the expression patterns of CD11a, CD11b, CD31, CD54, CD162, CD47, CD44, and CD29.

PMNs showed an increase of CD172a from the top to the cell layer fraction—in accordance with our in vivo data. The further increase of CD172a in the alveolar space was not detectable in vitro.

## 4. Discussion

PMN migration across the endothelium has been studied intensively by intravital microscopy, whereas transepithelial migration is mainly investigated in vitro or using an intestinal model [13] or by investigating intravascular and intra-alveolar PMNs [14]. So the later step of the migration is mainly under characterized, even though the epithelial tunnel, which the PMNs have to cross, is significantly longer (20 *μ*m or more) in contrast to the endothelial barrier of a few micrometers at most.

**Figure 7:**
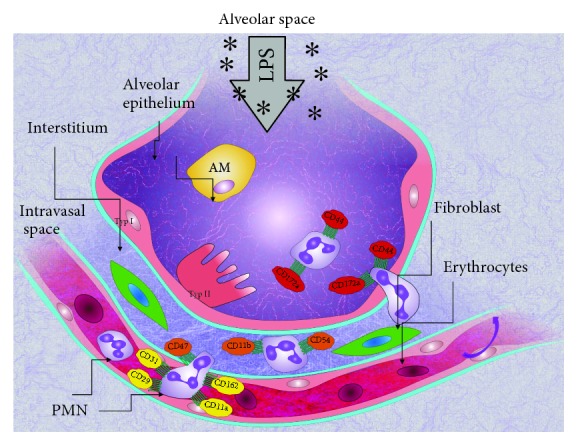
Schematic presentation of the investigated adhesion molecules on PMNs and their main site of action in pulmonary inflammation. The different molecules are displayed in the compartment of the highest expression, summarizing the results of the presented study.

With our technique, we are able to investigate every single migration step of the PMNs from the circulation, adherence to the endothelium, migration into the lung interstitium followed by a transepithelial migration into the alveolar space. To our knowledge, we are the first to determine the changes of the expression pattern of neutrophilic adhesion molecules on this way through the different compartments of the lung in vivo. Our results are summarized in Figure 7, presenting the predominant adhesion molecules in each compartment.

The leukocyte adhesion cascade is a tightly regulated process and the different steps are interconnected with each other. Therefore, the orchestration of PMN migration is regulated by chemokines, cytokines, adhesion molecules themselves, and also endogenous regulatory molecules (reviewed by [15, 16]). After the activation of integrins by chemokines or also for example by PSGL-1 ligation, integrins undergo major conformational changes resulting in presenting their ligand-binding site in the extracellular domains (reviewed by [17, 18]). The complexity of the interconnection becomes more apparent by the findings of Stadtmann et al. [19]. The authors identified an interaction between L-selectin and PSGL-1, which induced the activation of LFA-1 via different kinases. Also, neutrophils traffic for example to lymph nodes via high endothelial venules by employing PSGL-1 in concert with CD11b and CD11a [20]. In addition to the well-established pathways of selectin or chemokine-mediated integrin activation, there is increasing evidence that also Toll-like receptor signaling activates integrins [21, 22]. In the presented study, we did not investigate the interactions between the different analyzed adhesion molecules. This would be the subject of further investigations.

The results of the presented study complement the existing data and, for the first time, investigated the complete migration cascade of neutrophils throughout the lung including transendothelial and transepithelial migration.

The critical role of CD11a during transendothelial migration has long been established [23]. Within each migration step during the lung, the expression of CD11a decreased and, additionally, less PMNs showed the adhesion molecule on their surface, confirming the pivotal role for CD11a on the adhesion to the endothelium.

The other *β*_2_ integrin—besides CD11a—is CD11b. CD11b regulates intraluminal crawling on the endothelium [24]. Usually, these studies where done by intravital microscopy and only the initial steps of endothelial adhesion and migrating was investigated. In our study, the contact with the endothelium increased the expression of CD11b, but the migration through the lung interstitium even further enhanced the expression, highlighting a pivotal role for this integrin on transinterstitial migration. Pointing to this direction, Zen et al. demonstrated in vitro an important role for CD11b on firm adhesion of PMNs to the epithelium [25] and Kanse et al. identified matrix vitronectin as potential interstitial ligand [26].

The platelet and endothelial cell adhesion molecule (PECAM-1; CD31) is expressed on over 90% of the PMNs in each compartment of the lung. CD31 is known to play a major role in endothelial transmigration by the paracellular route [27]. Therefore, CD31 is expressed on both—the endothelium and PMNs interacting as adhesion molecule pair. Our study clearly indicates the highest expression of CD31 on circulating PMNs, indicating a distinct role of the adhesion molecule before adhering to the vascular endothelium. Pointing in the same direction, Ross et al. identified CD31 as a regulator of cells in the peripheral blood to migrate back into the bone marrow [28]. In the study of Ross et al., mice lacking PECAM-1 had higher level of circulating progenitor cells in the blood. Nevertheless, our data also reveals that the endothelial migration into the lung interstitium increased the expression of CD31 significantly, further highlighting the important role of the adhesion molecule on transendothelial migration.

In contrast, the expression of intercellular adhesion molecule- (ICAM-) 1 (CD54) increased significantly during the migration of the lung interstitium and is also highly expressed on PMNs in the alveolar space. A study on human volunteers supports our findings. Blood PMNs from patients showed lower levels of CD54 than PMNs that had been migrated into the bronchoalveolar lavage [14]. PMNs isolated from experimentally induced inflammatory lesions also had an enhanced expression of the adhesion molecule and the expression of CD54 was associated with fully primed neutrophils [29].

Confirming our findings with a high expression of CD54 on PMNs in the lung interstitium, the adhesion molecule moderates PMN motility by binding to fibrinogen [30].

P-Selectin glycoprotein ligand-1 (PSGL-1, CD162) is expressed on almost all PMNs in the circulation and decreases during the migration into the lung interstitium and again, after the migration into the alveolar space. The protein itself is highly expressed on circulating PMNs and already significantly reduced after the adherence to the pulmonary endothelium. This decline was further apparent after PMNs migrated into the lung interstitium and in the alveolar space. This is in accordance with the literature, where CD162 on circulating neutrophils binds P-selectin, resulting in the adherence of platelets [31–33] and therefore in capturing circulating PMNs. The PSGL-1 binds to L-selectin and this pathway triggers the activation of integrins on the inflamed endothelium and therefore initiates the slow rolling of neutrophils [19].

CD47—also known as the integrin-associated protein (IAP)—is ubiquitously expressed in all cells and has been reported to mediate PMN migration across the endothelium and epithelium after the initial *β*_2_ integrin-mediated adhesion [34, 35]. In CD47, knockout mice were less PMNs in the blood detectable and also in the BAL [36]. Therefore, it was unclear if the fewer circulating PMNs result in less migrated PMNs into the alveolar space or if there is a direct effect on transepithelial migration. Additionally, the alveolocapillary leakage as a marker for endothelial integrity was significantly reduced in knockout mice, pointing out the effect of the adhesion molecule on the endothelium. In the present study, around 40% of the circulating PMNs presented CD47 on their surface. CD47-positive PMNs significantly increased after the transendothelial migration and again—but not as prominent—after transepithelial migration. These findings are in line with newer publications, where a high expression of CD47 was accompanied with a lower rate of phagocytes [37]. The number of CD47 molecules on each PMN was already significantly more expressed on PMNs that adhered to the endothelium, remained stable in the lung interstitium, and decreased significantly after passing the epithelial barrier. Considering our data and the actual literature, we see the main effect of CD47 expressed on PMNs mainly on transendothelial migration.

Liu et al. found out that the exclusive inhibition of CD47 on neutrophils inhibited transepithelial migration in vitro [34]. At first sight, this is in contrast to our findings but it has to be considered that, to our knowledge, we are the first who describe the expression of the adhesion molecules through all compartments of the lung at once and are able to directly compare the different migration steps. Additionally, it is known that in vitro assays, which focus on one specific question from the whole organism, differ from in vivo findings, sometimes even fundamental [8].

CD47 functions also as a ligand for the signal regulatory protein (SIRP)*α*—CD172a—during neutrophil transmigration [38]. Since CD47 is also expressed from the pulmonary epithelium, this finding may explain many contradicting findings about the role of CD47 on transepithelial migration. In the presented work, the expression of CD172a is found on almost 100% of PMNs in the alveoli and the number of molecules on each PMN increased significantly during their migration through the different compartments of the lung, showing by far the highest expression on PMNs migrated into the alveolar space. These results suggest that CD172a on PMNs has its main impact on transepithelial migration. Emphasizing our findings, it has been shown that blocking CD172a with a specific inhibitor decreased transepithelial intestinal migration in vitro [38]. Also, PMN migration through CD47-deficient epithelial intestinal monolayers was significantly increased after the transfection with CD47 [34]. Since most of the studies on epithelial CD47 were performed with intestinal epithelium in vitro, Zemans et al. concluded in their review that the role of CD47 and therefore CD172a is not clear in pulmonary epithelium [39].

CD44 is a cell surface receptor, which has been shown to be involved in PMN adhesion to the endothelium. Mice lacking CD44 displayed a decrease in PMN adhesion and emigration after a chemotactic stimulus [40]. Also, depletion of CD44 and the removal of hyaluron—the main ligand of CD44—from the vascular endothelium impaired neutrophil migration [40]. Blocking CD44 after the administration of LPS detached adherent PMNs to the liver sinusoid [41]. These findings support our results of the presented study, where only 40% of the circulating PMNs have CD44 on their surface, whereas the adherence to the endothelium increased the amount of over 80%. Additionally, the adherence provoked on PMNs presenting CD44 that the molecule expression itself on the PMN surface more than doubled, emphasizing the importance of the adhesion molecule on endothelial adherence and transmigration. Passing the epithelial barrier from the lung interstitium into the alveolar space further increased the expression of CD44 to almost 100% of migrated PMNs and also the expression of the molecule itself triplicated from intravasal to intra-alveolar, further highlighting a pivotal role of CD44 on transepithelial migration. An effect of CD44 on intestinal epithelial PMN migration has been shown [42], but to our knowledge, we are the first to demonstrate this effect in the pulmonary epithelium.

Another alternate neutrophil adhesion molecule that is involved in PMN transmigration is CD29 (*β*_1_-integrin). CD29 has also been shown to be involved in transendothelial migration [43]. Our results show the highest percentage of CD29-positive PMNs and also the amount of surface expression on PMNs—both—on circulating PMNs. There was a significant reduction after the adherence to the endothelium, but consistent with the literature, a significant increase of the expression of CD29 was detectable after passing the transendothelial barrier. In the alveolar space, less PMNs expressed the adhesion molecule and the number of molecules on each PMN was additionally significantly decreased, indicating a minor role of CD29 on transepithelial migration. In a mouse model of LPS inhalation, blocking CD29 reduced PMN accumulation in the capillaries of the lung and the alveolar space [44]. The decreased number of PMNs after inhibiting CD29 in the capillaries is consistent with our findings, where the expression was the highest intravasal. The reduced PMN influx into the alveolar space can be explained by the increased expression of CD29 in the lung interstitium, and after blocking, these PMNs were arrested in the lung and not able to migrate any further. In the study from Ridger et al., PMNs in the different compartments of the lung were determined by electron micrographs from five to six sections for each group. CD29 is used from pneumocytes to interact with fibronectin, a matrix protein of the lung [45], supporting further our findings from a critical role of CD29 on interstitial PMN migration.

The expression profile of each adhesion molecule on the PMN populations of the different compartments of the lung changed while the cells migrated through the lung. These changes of the expression pattern can be possible by, for example, shedding of the adhesion molecules leading to a reduction of the expression [46]. Internalization of adhesion molecules is also described [47], which may allow a later externalization of the adhesion molecule for another migration step. This might explain how the distinct expression of CD29 is possible: high expression in the vascular compartment, a reduced pattern when PMNs were attached to the endothelium, an increase of expression again in the lung interstitium followed by another reduction on PMNs migrated into the alveolar space. The mechanisms behind the changes of the expression patterns of the adhesion molecules remain elusive and are part of further investigations. Also, they may differ for each adhesion molecule.

The affinity state of integrins play an important role for transmigration. CD11a, for example, is known to be able to shift from a low to intermediate and also high affinity state [48, 49]. The different affinity states have unequal roles. The shift from low to intermediate affinity state of CD11a supports rolling on the endothelium, whereas the high affinity is associated with shear resistant leukocyte arrest and both affinity states and especially the shifts are important for leukocyte transmigration on inflamed endothelium [50].

## 5. Conclusion

Modulation of PMN migration is one of the challenging but at the same time most promising target in developing therapeutic options. We show for the first time the shift of different adhesion molecules on PMNs during the complete migration process through the different compartments of the lung. This new knowledge crucially increases the pathophysiological understanding of PMN migration leading to therapeutic options of acute pulmonary inflammation.

## Data Availability

The data used to support the findings of this study are available from the corresponding author upon request.

## Supplementary Materials

Supplementary Materials Supplemental Figure 1: isotype controls of adhesion molecule antibodies expression by immunofluorescence. Matching IgG controls of the antibodies marking CD29, CD31, and CD54 were used. Images are representatives of four experiments with similar results (original magnification, ×63). Supplemental Figure 2: gating process. Typical dot blots in the forward/sideward scatter of cells in the blood, lung, and bronchoalveolar lavage (BAL) are shown. (A) The gating process in the lung tissue with differentiation of leukocytes (CD45+) and the following further distribution in PMNs adherent to the endothelium (7/4+ and Gr-1+ cells) and PMNs in the interstitium of the lung (7/4+ and Gr-1-). (B) The gating process of one adhesion molecule on PMNs in the BAL is demonstrated. (C) PMNs were gated in the dot plot and first identified as CD45+. This CD45+ population was further gated as GR-1+ and 7/4+. The identified PMN cloud was further evaluated on the expression of the distinct adhesion molecule with PE- staining. Supplemental Figure 3: schematic presentation of the in vitro transmigration assay. In the lower chamber is the stimulant fMLP (n-formylmethionyl-leucyl-phenylalanine), which attracts human PMNs to migrate through the pulmonary epithelial monolayer (H441 cells). Supplemental Figure 4: mean fluorescence intensity (MFI). Each peak of the histogram resembles one PMN population. The more fluorescent antibody is bound on the PMN surface, the higher the MFI.
