# Supplementary material for: How Adhesion Molecule Patterns Change While Neutrophils Traffic through the Lung during Inflammation
Source: Mediators Inflamm. 2019 Feb 28;2019:1208086. doi: 10.1155/2019/1208086 (PMC6421765; doi:10.1155/2019/1208086)

Supplemental figure 1: Isotype-controls of adhesion molecule antibodies expression by immunofluorescence. Matching IgG-controls of the antibodies marking CD29, CD31, and CD54 were used. Images are representatives of four experiments with similar results (original magnification, x63).

Supplemental figure 2: Gating process. Typical dot blots in the forward / sideward scatter of cells in the blood, lung and bronchoalveolar lavage (BAL) are shown (**A**). The gating process in the lung tissue with differentiation of leukocytes ($CD45^+$) and the following further distribution in PMNs adherent to the endothelium ($7/4^+$ and $Gr\text{-}1^+$ cells), and PMNs in the interstitium of the lung ($7/4^+$ and $Gr\text{-}1^-$) (**B**). The gating process of one adhesion molecule on PMNs in the BAL is demonstrated (**C**). PMNs were gated in the dot plot and first identified as $CD45^+$. This $CD45^+$ population was further gated as $GR\text{-}1^+$ and $7/4^+$. The identified PMN cloud was further evaluated on the expression of the distinct adhesion molecule with PE- staining.

Supplemental figure 3: Schematic presentation of the in vitro transmigration assay. In the lower chamber is the stimulant fMLP (n-formylmethionyl-leucyl-phenylalanine), which attracts human PMNs to migrate through the pulmonary epithelial monolayer (H441 cells).

Supplemental figure 4: Mean fluorescence intensity (MFI). Each peak of the histogram resembles one PMN population. The more fluorescent antibody is bound on the PMN surface, the higher the MFI.

Supplemental figure 1

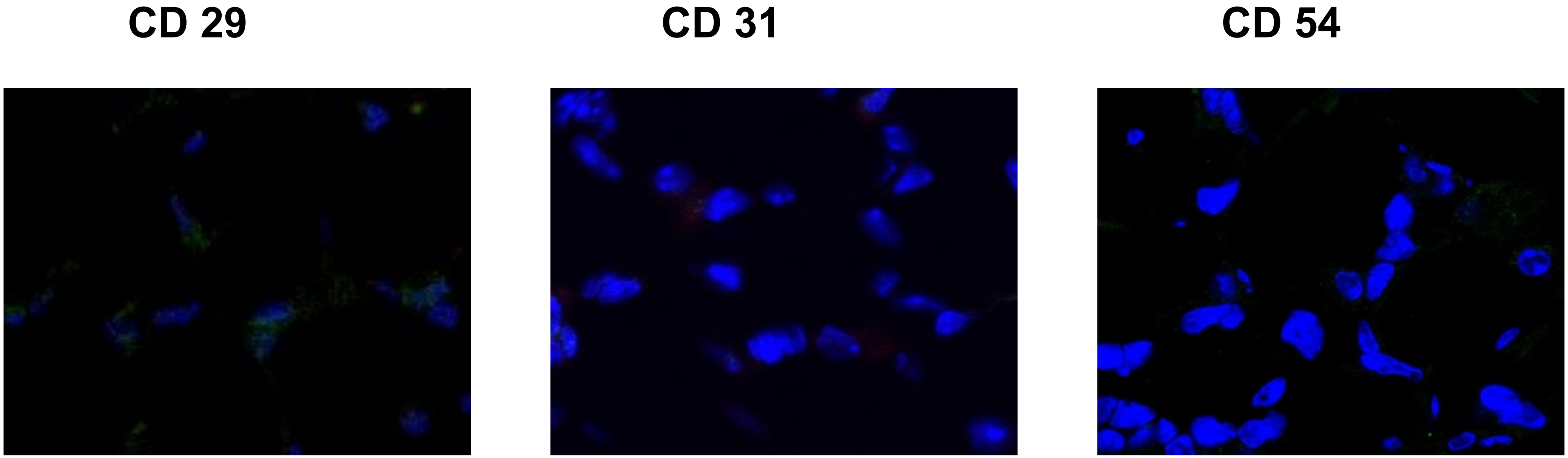

Supplemental figure 2

A

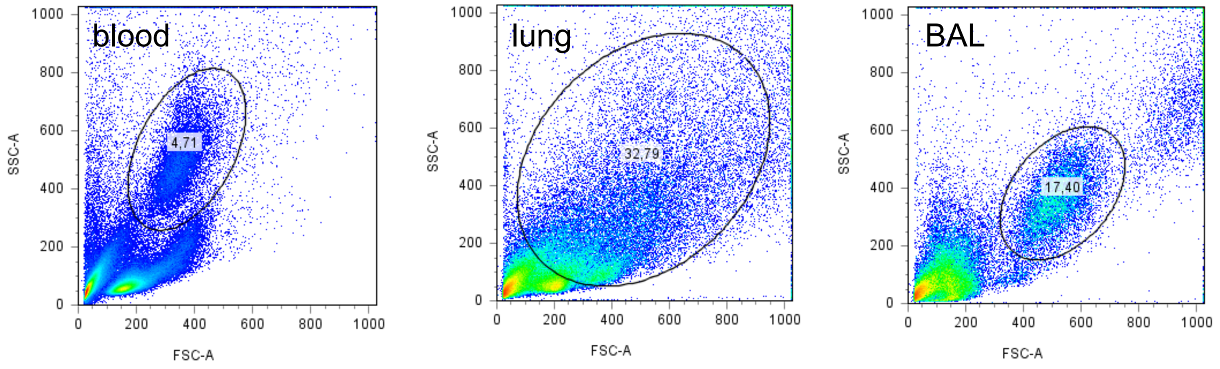

B

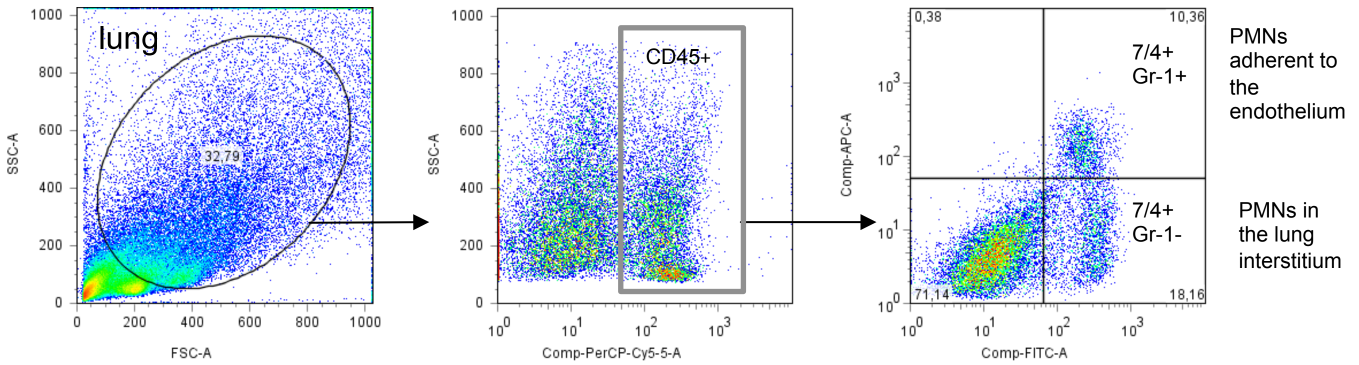

C

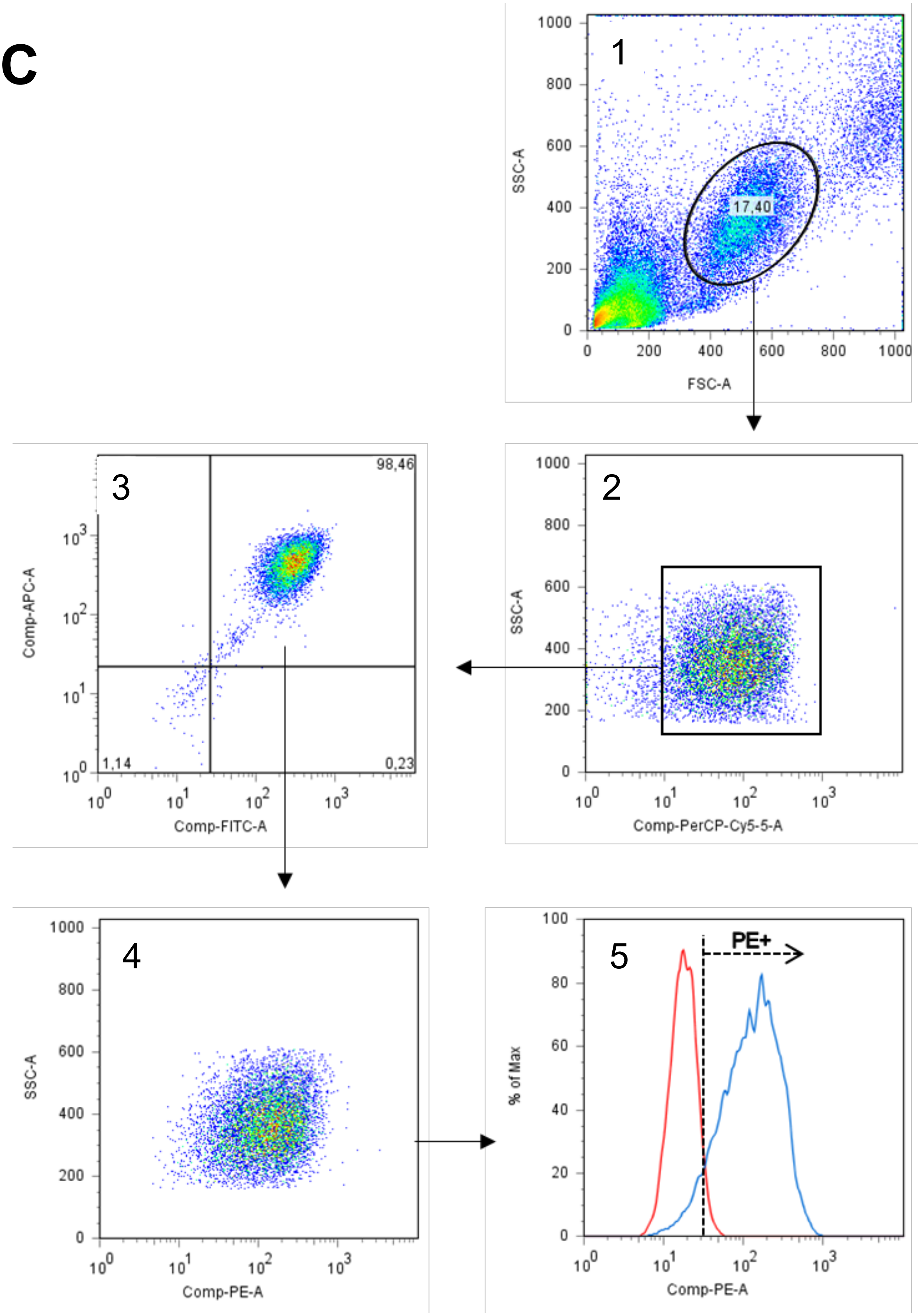
1
17,40
SSC-A
FSC-A
2
SSC-A
Comp-PerCP-Cy5-5-A
3
98,46
1,14
0,23
Comp-APC-A
Comp-FITC-A
4
SSC-A
Comp-PE-A
5
PE+
% of Max
Comp-PE-A

Supplemental figure 3

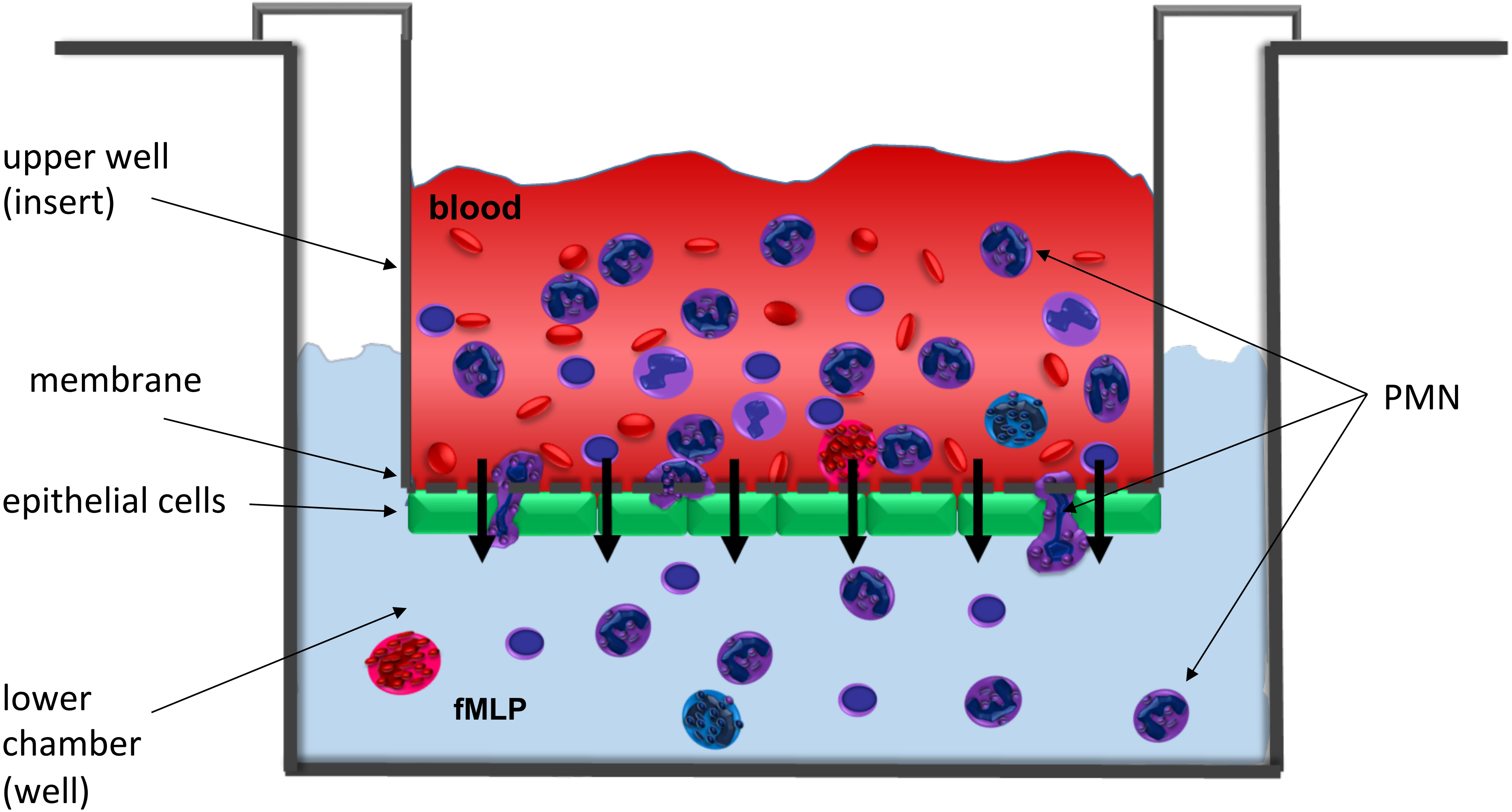

Supplemental figure 4

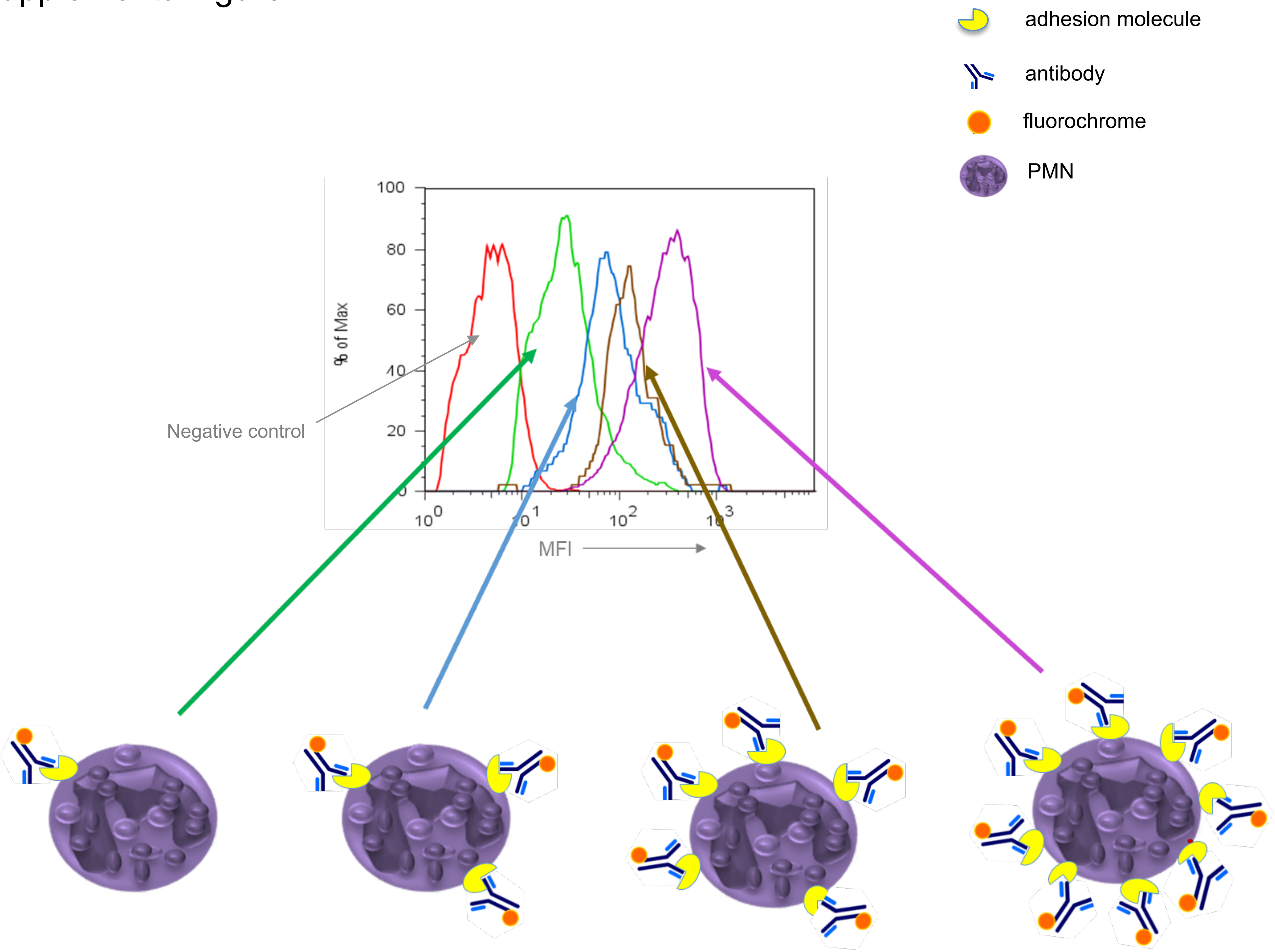

Supplement: Supplementary Materials — Supplemental Figure 1: isotype controls of adhesion molecule antibodies expression by immunofluorescence. Matching IgG controls of the antibodies marking CD29, CD31, and CD54 were used. Images are representatives of four experiments with similar results (original magnification, ×63). Supplemental Figure 2: gating process. Typical dot blots in the forward/sideward scatter of cells in the blood, lung, and bronchoalveolar lavage (BAL) are shown. (A) The gating process in the lung tissue with differentiation of leukocytes (CD45+) and the following further distribution in PMNs adherent to the endothelium (7/4+ and Gr-1+ cells) and PMNs in the interstitium of the lung (7/4+ and Gr-1-). (B) The gating process of one adhesion molecule on PMNs in the BAL is demonstrated. (C) PMNs were gated in the dot plot and first identified as CD45+. This CD45+ population was further gated as GR-1+ and 7/4+. The identified PMN cloud was further evaluated on the expression of the distinct adhesion molecule with PE- staining. Supplemental Figure 3: schematic presentation of the in vitro transmigration assay. In the lower chamber is the stimulant fMLP (n-formylmethionyl-leucyl-phenylalanine), which attracts human PMNs to migrate through the pulmonary epithelial monolayer (H441 cells). Supplemental Figure 4: mean fluorescence intensity (MFI). Each peak of the histogram resembles one PMN population. The more fluorescent antibody is bound on the PMN surface, the higher the MFI. [file 1208086.f1.pdf]
